# HBV and HCV Burden in a Greek Hospital Population (2018–2024): Trends and Correlates of HBsAg and Anti-HCV Positivity

**DOI:** 10.3390/pathogens15030342

**Published:** 2026-03-23

**Authors:** Nikolaos Georgiadis, Christina Seitopoulou, Maria Kimouli, Theodoros N. Sergentanis, Apostolos Beloukas, Georgina Tzanakaki

**Affiliations:** 1 Laboratory Surveillance of Infectious Diseases, Department of Public Health Policy, School of Public Health, University of West Attica, 11521 Athens, Greece; 2 Microbiology Laboratory, “Agios Panteleimon” General Hospital, 18554 Piraeus, Greece; 3 Department of Biomedical Sciences, School of Health Sciences, University of West Attica, 12243 Athens, Greece

**Keywords:** hepatitis B, hepatitis C, viral infection, disease burden, epidemiological data, prevalence, Greece

## Abstract

**Background**: Hepatitis B and C remain a major public health challenge in Greece, particularly amid demographic shifts, migration, and evolving socioeconomic conditions. Updated epidemiological data are essential to guide public health planning and prevention strategies. **Methods**: A repeated cross-sectional study was conducted among adults (n = 36,085) attending the General Hospital of Nikaia “Agios Panteleimon”, Piraeus, Greece, from 2018 to 2024. Participants consisted of inpatients and outpatients, including recognized high-risk groups. Serological markers assessed current hepatitis B infection (HBsAg) and past or recent hepatitis C exposure (anti-HCV). Associations were examined using univariate and multivariate logistic regression, reporting adjusted odds ratios (aORs) and 95% confidence intervals (CIs). **Results**: Overall prevalence was 4.65% for HBsAg (n = 1677) and 6.6% for anti-HCV (n = 2378). Females had significantly lower odds compared to males for both markers (HBsAg aOR = 0.24, anti-HCV aOR = 0.77, both *p* < 0.001). Anti-HCV prevalence declined with age, with the ≥70 group showing the lowest odds (aOR = 0.24, *p* < 0.001). For HBsAg, older age groups also showed reduced odds, particularly ages 60–69 (aOR = 0.49, *p* < 0.001) and ≥70 (aOR = 0.75, *p* = 0.005). Compared to Attica region, most regions had significantly lower odds of both infections, including Thrace (HBsAg aOR = 0.08; anti-HCV aOR = 0.32, both *p* < 0.001), Crete (HBsAg aOR = 0.13; anti-HCV aOR = 0.35, both *p* < 0.001), and Macedonia (HBsAg aOR = 0.37; anti-HCV aOR = 0.64, both *p* < 0.001). Compared to 2018, the odds were markedly higher in 2023 and peaked in 2024 for both infections (anti-HCV aOR = 1.78; HBsAg aOR = 3.10, both *p* < 0.001 for 2024). High-risk social groups demonstrated substantially elevated odds of anti-HCV (aORs 3.9–5.51, all *p* < 0.001), but had lower odds of HBsAg (aORs 0.32–0.60, all *p* ≤ 0.001). **Conclusions**: Increasing prevalence trends, regional disparities, and pronounced differences among vulnerable groups highlight the urgent need for strengthened screening, vaccination, and targeted hepatitis B and C prevention strategies, particularly among healthcare-attending and high-risk populations in Greece.

## 1. Introduction

Viral hepatitis, including infections caused by hepatitis B virus (HBV) and hepatitis C virus (HCV), remains a major global public health problem, contributing substantially to morbidity and mortality worldwide [1]. Chronic HBV infection affects an estimated 256 million individuals and represents a leading cause of liver cirrhosis and hepatocellular carcinoma (HCC), while approximately 57 million people are living with chronic HCV infection globally, with ongoing transmission sustaining disease burden despite major therapeutic advances [2,3].

Accurate epidemiological data are essential for quantifying disease burden and informing public health strategies. Serological markers such as hepatitis B surface antigen (HBsAg) and antibodies against hepatitis C virus (anti-HCV) are widely used to estimate the prevalence of chronic HBV infection and exposure to HCV at the population level [4]. However, in countries of low or intermediate endemicity, prevalence estimates are often derived from heterogeneous sources or selected populations, limiting their generalizability [4].

In Greece, estimates of HCV prevalence in the general population vary considerably. Available evidence suggests that anti-HCV seroprevalence ranges approximately from 0.5% to 2%, reflecting substantial heterogeneity across geographic regions, population subgroups, and study designs, as well as reliance on data from registries, blood donors, regional surveys, and high-risk populations rather than uniform population-based sampling [5]. This variability highlights the uncertainty regarding the true burden of HCV infection at the national level.

Similarly, Greece is currently classified as a low-endemicity country for HBV, yet the epidemiology of HBV infection is characterized by marked regional and population heterogeneity [6]. Persistent high-prevalence clusters have been described in specific regions, alongside temporal changes in transmission patterns and an increasing contribution of migrant populations from higher-endemicity regions [7]. These features complicate accurate estimation of HBV burden and underscore the limitations of extrapolating national prevalence from localized or clinically based datasets [8].

Taken together, the heterogeneity and uncertainty surrounding existing estimates of HBV and HCV infection in Greece indicate the need for contemporary, population-relevant seroepidemiological data using standardized biomarkers. Accordingly, the present study aimed to estimate the seroprevalence of HBsAg and anti-HCV and to examine associated demographic, clinical, geographic, and temporal factors in a large hospital-based population over a seven-year period (January 2018–December 2024).

## 2. Materials and Methods

Reporting followed the Strengthening the Reporting of Observational Studies in Epidemiology (STROBE) statement for cross-sectional studies, and a completed STROBE checklist is provided as Supplementary Material [9].

The study population comprised both inpatients and outpatients who attended the General Hospital of Nikaia “Agios Panteleimon”, Piraeus, Greece, for any reason, either as an inpatient or an outpatient, from January 2018 to December 2024, including individuals from the general population as well as recognized high-risk groups for viral hepatitis. Specifically, the high-risk groups included: (1) former and current intravenous drug users, (2) men who have sex with men (MSM), (3) individuals of Roma (Gypsy) origin, (4) refugees, (5) migrants, (6) incarcerated individuals, and (7) sex workers. All adult participants (over 18 years of age) with available HBV and HCV serological testing from 2018 to 2024 were included (n = 36,085). Each participant was uniquely identified using a patient-specific identification number, and duplicate records were excluded, ensuring that each individual was included only once in the analysis. In the absence of a national vaccination registry in Greece, hepatitis B vaccination status was obtained through self-report. Among participants reporting prior hepatitis B vaccination, medical records and available laboratory results were reviewed to exclude individuals with evidence of previous hepatitis B infection.

Blood samples were collected via venipuncture using serum separator tubes. Following collection, samples were centrifuged at 4000 rpm for 12 min and analyzed within two hours. Serological testing for HBsAg and anti-HCV was performed using the ABBOTT Architect i2000 SR analyzer (Abbott Diagnostics, Athens, Greece), based on chemiluminescent microparticle immunoassay (CMIA) technology. Commercially available assays were used in accordance with standard laboratory procedures and the manufacturer’s instructions. This study was conducted in accordance with ethical standards and received approval from the Scientific Council and the Ethics Committee of the General Hospital of Nikaia-Piraeus “Agios Panteleimon” (approval code: 14124/27-03-23, approval date: 23 March 2023). A supplemental approval was also granted by the same institution (approval code: 45130/04-10-2023, approval date: 4 October 2023). Furthermore, ethical approval was obtained from the Research Ethics and Ethics Committee (REEC) of the University of West Attica (UNIWA) (approval code: 111095/14-11-2023, approval date: 16 January 2024).

Data collection covered the period from January 2018 to December 2024. Given the inclusion of personal health data, all required documentation was submitted to the aforementioned institutional bodies, and appropriate authorizations were obtained prior to data acquisition and analysis.

### Statistical Analysis

Descriptive statistics were used to summarize demographic, geographic, clinical, and social characteristics of the study population. Categorical variables were reported as frequencies and percentages.

Logistic regression analysis was performed to examine associations between participant characteristics and serological evidence of HBsAg and anti-HCV positivity, respectively. For each outcome, univariate and multivariate logistic regression models were fitted. Variables were initially screened in univariate models, and those with a *p*-value < 0.05 were considered for inclusion in the multivariate analysis. Unadjusted odds ratios (ORs) with 95% confidence intervals (CIs) and corresponding *p*-values were obtained from univariate analyses, while adjusted odds ratios (aORs) with 95% CIs were estimated from multivariate models.

For the HBV analysis, regression models were restricted to individuals who self-reported not being vaccinated against hepatitis B, in order to focus on those presumed at risk of infection. However, vaccination status was based on self-report and may be subject to misclassification. Only one variable (i.e., sex) had missing data, affecting 50 individuals (0.14% of the total sample). All other variables were complete. Given the very small proportion of missing data, cases with missing sex were omitted from analyses where sex was used as a covariate.

The level of statistical significance was set at 0.05. Statistical analysis was performed with STATA/SE version 16 (Stata Corp., College Station, TX, USA).

## 3. Results

### 3.1. Study Population Characteristics and Overall Seropositivity

A total of 36,085 individuals participated in the study examining the prevalence of HbsAg and anti-HCV in Greece. Of these, 59.9% (n = 21,601) were male, 40% (n = 14,434) were female, and the gender was missing for 0.14% (n = 50). The age distribution was relatively even within each group, ranging from 18 to 70 years and older, comprising between 16.5% and 16.8% of the sample. Specifically, participants aged 18–29 accounted for 16.7%; 30–39 age group for 16.8%; 40–49 for 16.8%; 50–59 for 16.6%; 60–69 for 16.5%; and individuals aged 70 and above for 16.5%.

In terms of nationality, 59.9% (n = 21,621) were Greek while 40.1% (n = 14,464) were non-Greek; the largest foreign-born groups included individuals from Albania (3.9%), Afghanistan (3.8%), Pakistan (2.6%), and Syria (2.6%), followed by smaller groups from Somalia, Iraq, Iran, Morocco, Ethiopia, Egypt, Bangladesh, the Philippines, Georgia, Ukraine, Russia, Senegal, India, Moldova, China, South Africa, Turkey, Bulgaria, Romania, and Palestine.

The majority of participants resided in Attica, accounting for 50.7% of the sample. Other regions with notable representation included the Peloponnese (10.2%), Thessaly (8.0%), Macedonia (6.1%), the Cycladic and Dodecanese Islands (each with 6.9%), and Crete (2.8%). Smaller proportions were reported from Thrace (1.9%), the Sporades (1.9%), the Ionian Islands (1.9%), and Euboea (2.6%).

In terms of social and vulnerable groups, 60% of participants were not part of any social minority or high-risk group; the most represented high-risk groups included Roma (11.1%), incarcerated individuals (10.7%), substance users (4.5%), refugees (4.6%), immigrants (4.5%), sex workers (2.8%), and MSM (1.6%). Data collection was distributed relatively evenly across a seven-year period, with the percentage of individuals tested or diagnosed in each year ranging from 13.8% in 2018 to 15% in 2019, while the following years showed comparable proportions, such as 14.0% in 2020, 13.8% in 2021, 14.9% in 2022, 14.3% in both 2023 and 2024, respectively.

Vaccination coverage for hepatitis B was nearly balanced, with 48.1% of participants reporting being vaccinated and 51.9% not vaccinated. Underlying health conditions were reported by 22.9% of the sample; the most prevalent were lipid disorders (9.4%), diabetes mellitus (5.1%), and autoimmune diseases (3.8%), followed by neurological diseases (2.8%) and cardiovascular disease (1.8%). The remaining 77.1% of participants reported no known underlying conditions.

Serological testing showed that 4.6% (n = 1677) of participants were positive for HBsAg, indicating current HBV infection. HBeAg, a marker of infectivity, was found in 2.6% of individuals, and 2.1% tested positive for anti-HBe. Regarding hepatitis C, 6.6% (n = 2378) of participants tested positive for anti-HCV antibodies, reflecting recent or past infection with HCV, while the remaining 93.4% were negative.

### 3.2. Factors Associated with Anti-HCV Seropositivity

The univariate and multivariate models revealed various significant associations with anti-HCV positivity (Table 1). Gender was a significant predictor of anti-HCV positivity. Compared to males, females had significantly lower odds in both the univariate (OR: 0.78, 95% CI: 0.71–0.85, *p* < 0.001) and multivariate analysis (aOR = 0.77, 95% CI: 0.70–0.84, *p* < 0.001).

Age showed a strong and consistent inverse association with anti-HCV seropositivity. Using the 18–29 age group as the reference category, all older age groups exhibited lower odds of anti-HCV positivity in the multivariable model, with all associations reaching statistical significance. The effect was particularly pronounced among those aged 50–59 (aOR: 0.52, 95% CI: 0.45–0.60), 60–69 (aOR = 0.41, 95% CI: 0.35–0.48) and ≥70 years (aOR = 0.24, 95% CI: 0.20–0.29), with *p*-values < 0.001. These results were largely consistent with the univariate analysis.

Nationality in broad terms (Greek vs. foreign) was not significantly associated with anti-HCV positivity in the unadjusted model (*p* = 0.884). However, analysis by specific country of origin revealed several important findings. Individuals from Ethiopia had over twice the odds of anti-HCV seropositivity compared to Greeks (aOR = 2.15, 95% CI: 1.63- 2.83, *p* < 0.001), and elevated odds were also observed among participants from Albania (aOR = 1.43, *p* < 0.001), Russia (aOR = 1.51, *p* = 0.041), and Bulgaria (aOR = 1.51, *p* = 0.037). Conversely, participants from Syria (aOR = 0.52, *p* < 0.001), Somalia (aOR = 0.59, *p* = 0.003) and Iraq (aOR = 0.69, *p* = 0.032) had significantly lower odds compared to Greeks. Nationality-specific associations were largely consistent between the univariate and multivariate models. However, three countries that showed significant associations only after adjustment had not reached significance in the univariate analysis: Bulgaria (OR = 1.27, *p* = 0.207), Russia (OR = 1.19, *p* = 0.362), and Iraq (OR = 0.75, *p* = 0.081).

Region of residence was also significantly associated with anti-HCV status, with the associations observed in the univariate model remaining significant after multivariate adjustment. Specifically, in comparison with Attica region, residence in regions such as Macedonia (aOR = 0.64), Thrace (aOR = 0.32), Peloponnese (aOR = 0.81), Ionian islands (aOR = 0.57), Cycladic Islands (aOR = 0.35), Dodecanese Islands (aOR = 0.32), Sporades (aOR = 0.33), Euboea (aOR = 0.34) and Crete (aOR = 0.35), was associated with substantially lower odds (all *p* ≤ 0.004). On the other hand, Thessaly was associated with slightly increased odds (aOR = 1.19, *p* = 0.017).

In terms of temporal trends, the odds of anti-HCV positivity increased in more recent years. Compared to 2018, significantly higher odds were observed in 2020 (aOR = 1.2, 95% CI: 1.01–1.42, *p* = 0.037), 2023 (aOR = 1.38, 95% CI: 1.17–1.63, *p* < 0.001) and 2024 (aOR = 1.78, 95% CI: 1.52–2.09, *p* < 0.001), suggesting a possible rising trend in newly identified cases or shifts in testing coverage. These patterns were also consistent with the univariate findings, except for the year 2020 (OR: 1.17, 95% CI: 0.99–1.38, *p* = 0.058). Temporal trends in anti-HCV seropositivity, overall and stratified by sex, are presented in Supplemental Figures S1 and S2.

While hepatitis B vaccination appeared protective in the unadjusted analysis (OR = 0.40, *p* < 0.001), the association was no longer statistically significant in the adjusted model (aOR = 1.27, *p* = 0.087).

Certain underlying medical conditions showed important associations. After adjustment, autoimmune diseases (aOR = 1.75, *p* < 0.001), neurological diseases (aOR = 2.02, *p* < 0.001), and cardiovascular disease (aOR = 2.01, *p* = 0.001) were independently associated with higher odds of anti-HCV seropositivity. In contrast, individuals with diabetes mellitus (aOR = 0.63, *p* = 0.003) and lipid disorders (aOR = 0.68, *p* < 0.001) had significantly lower odds. All of the aforementioned associations, except for neurological disorders (OR: 0.95, 95% 0.74–1.22, *p* = 0.68), were evident from the univariate analysis and were retained in the multivariate model.

Finally, the analysis revealed strikingly elevated odds of anti-HCV positivity among individuals belonging to vulnerable social groups in the univariate analysis that persisted in the multivariate model. Specifically, compared to the general population, significantly higher odds were observed among ex and current substance users (aOR = 4.68, 95% CI: 3.45–6.32), MSM (aOR = 5.51, 95% CI: 3.85–7.89), Roma (aOR = 4.98, 95% CI: 3.79–6.55), refugees (aOR = 5.27, 95% CI: 3.91–7.09), immigrants (aOR = 3.9, 95% CI: 2.87–5.31), incarcerated individuals (aOR = 5.41, 95% CI: 4.11–7.12), and sex workers (aOR = 4.59, 95% CI: 3.28–6.41), all with *p*-values < 0.001.

### 3.3. Factors Associated with HBsAg Seropositivity

Several factors were significantly associated with HBsAg seropositivity in both univariate and multivariable analyses. Full details of these associations are presented in Table 2. Regarding HBsAg seropositivity, sex was a strong predictor, with females showing significantly lower odds of infection compared to males (aOR = 0.24, 95% CI: 0.21–0.27, *p* < 0.001), consistent with the univariate results. Age was also significantly associated with HBsAg status in selected groups. In the univariate analysis, individuals aged 60–69 showed significantly lower odds of HBsAg seropositivity compared to the age group 18–29 (OR = 0.54, 95% CI: 0.43–0.66, *p* < 0.001). However, in the multivariate model, those aged 50–59 (aOR = 0.75, *p* = 0.008), 60–69 (aOR = 0.49, *p* < 0.001), and ≥70 (aOR = 0.75, *p* = 0.005) had significantly lower odds compared to those aged 18–29 years. Overall nationality (Greek vs. foreign) was associated with slightly lower odds of HBsAg positivity among foreign nationals in the univariate model (OR = 0.90, *p* = 0.039); however, specific country-level data provided more insight. After adjustment, individuals from Albania (aOR = 1.91, *p* < 0.001) and Pakistan (aOR = 1.55, *p* = 0.002) had significantly higher odds compared to Greek origin. In contrast, individuals from Moldova (aOR = 0.24, *p* = 0.002), Georgia (aOR = 0.49, *p* = 0.01), Philippines (aOR = 0.57, *p* = 0.034), Bangladesh (aOR = 0.59, *p* = 0.046), Iraq (aOR = 0.58, *p* = 0.015) and China (aOR = 0.47, *p* = 0.041) had significantly lower odds. These patterns were largely consistent with the univariate model. In contrast, individuals originating from South Africa (OR = 0.54, *p* = 0.049) and Iran (OR = 0.65, *p* = 0.039) showed statistically significant associations with HBsAg positivity only in the univariate analysis, which did not persist after multivariable adjustment. Significant geographical variation in HBsAg seropositivity was observed. Compared to residents in Attica, those living in all other regions except Peloponnese had markedly lower odds both in the univariate and multivariate models (all aOR: 0.08–0.37 *p* < 0.001). The lowest adjusted odds were seen in Thrace (aOR = 0.08, *p* < 0.001), Crete (aOR = 0.13, *p* < 0.001), and the Sporades (aOR = 0.09, *p* < 0.001).

In terms of temporal trends, the odds of HBsAg positivity were significantly higher in more recent years compared to 2018. The adjusted odds increased progressively in 2022 (aOR = 1.27, *p* = 0.029), 2023 (aOR = 1.51, *p* < 0.001), and 2024 (aOR = 3.10, *p* < 0.001)—all consistent with the univariate results, indicating a rising detection or true prevalence in recent years. Temporal trends in HBsAg seropositivity among unvaccinated individuals, overall and stratified by sex, are presented in Supplemental Figures S3 and S4. Several underlying medical conditions were significantly associated with HBsAg positivity. Individuals with diabetes mellitus (aOR = 2.72, *p* < 0.001) and neurological conditions (aOR = 2.09, *p* < 0.001) had increased odds for HBsAg positivity, while those with cardiovascular disease (aOR = 0.59, *p* = 0.002), autoimmune diseases (aOR = 0.47, *p* = 0.001) and lipid disorders (aOR = 0.45, *p* < 0.001) had reduced odds. All the aforementioned associations regarding medical conditions were apparent in the univariate analysis, except for cardiovascular disease (OR: 0.88, *p* = 0.427).

Contrary to patterns observed with anti-HCV, individuals belonging to vulnerable or minority social groups had significantly lower odds of HBsAg seropositivity compared to the general population both in the univariate and multivariate analysis. This included ex and current substance users (aOR = 0.55), MSM (aOR = 0.32), Roma (aOR = 0.53), refugees (aOR = 0.59), immigrants (aOR = 0.57), incarcerated individuals (aOR = 0.60), and sex workers (aOR = 0.60), all with *p*-values ≤ 0.001.

## 4. Discussion

In this large cross-sectional study conducted in a single hospital in Greece (n = 36,085), current HBV infection (HBsAg positivity) was identified in 4.6% of participants, while 6.6% were anti-HCV positive, indicating past or current HCV exposure. In adjusted analyses, anti-HCV seropositivity was lower among females and declined with increasing age, but was markedly higher among several vulnerable groups, including substance users, MSM, Roma, refugees, immigrants, incarcerated individuals, and sex workers (aORs approximately 3.9–5.5). Additional heterogeneity was observed by country of origin and region of residence, with higher odds among individuals originating from Albania and Ethiopia and lower odds among those from Syria, Somalia, and Iraq, as well as substantially lower odds in most regions outside Attica. Anti-HCV positivity was also more likely in recent testing years, particularly in 2023–2024, compared with 2018. For HBsAg, females again had substantially lower odds of HBsAg positivity, older age groups generally showed reduced adjusted odds, and prevalence varied by country of origin and geography, with higher odds among individuals from Albania and Pakistan and markedly lower odds outside Attica; notably, unlike HCV, being part of vulnerable groups was associated with lower adjusted odds of HBsAg positivity.

Compared with prior Greek estimates, the HBsAg prevalence in our hospital-based sample (4.65%) is substantially higher than what has typically been reported for the general population, where Greece is generally characterized as middle endemicity with HBsAg prevalence in the general population estimated 1–2% in the latest European Centre for Disease Prevention and Control (ECDC) reports [10], while markedly higher levels have been documented in specific subpopulations such as migrants from higher-endemicity countries. Likewise, the anti-HCV prevalence we observed (6.6%) exceeds most general-population estimates for Greece-roughly 2.2% in the years 2005–2015 in the latest ECDC report [11]. Taken together, these comparisons suggest that the higher seroprevalence in this study could likely reflect the non-representative, single-hospital sampling frame and the over-representation of vulnerable/high-risk groups, rather than a direct shift in population-wide prevalence. Given the hospital-based design and inclusion of high-risk groups, our findings should not be interpreted as representative of the general population in Greece. Rather, they reflect the epidemiological profile of a large healthcare-attending population, which may differ from community-based estimates.

Females were independently associated with lower odds of both HBsAg and anti-HCV seropositivity. Similar differences have been reported previously, with males consistently shown to have a higher prevalence of HBV exposure and chronic infection, as well as a greater risk of HBV-related liver disease, reflecting a combination of sex hormone-mediated biological effects and gender-related differences in exposure [12]. For HCV, higher prevalence among males has also been described in population-based and clinical studies, with females more likely to clear infection and experience slower disease progression [13]. Given that anti-HCV antibodies persist after viral clearance, sex differences in seroprevalence are unlikely to be explained by biological clearance mechanisms alone; instead, they are more plausibly attributable to differential lifetime exposure to major transmission routes, as reflected by higher prevalence of risk behaviors and blood-borne co-infections among men [14].

In our study, anti-HCV positivity decreased with age. However, age-specific patterns vary considerably across countries and testing settings. In European data, some general-population surveys report higher prevalence among older individuals, consistent with cumulative lifetime exposure. In contrast, studies conducted in healthcare-based or risk-enriched settings often show higher positivity among younger or middle-aged adults. These differences likely reflect variation in testing practices and risk distribution across age groups, particularly the greater representation of individuals with ongoing or recent exposures in certain screening contexts [15]. HBsAg showed a similar pattern in our analysis, with lower odds of positivity observed in several older age groups. Comparable findings have been reported in previous seroepidemiological studies and are generally attributed to cohort effects and differences in infection acquisition over time, rather than to age-related biological mechanisms [16].

Although we observed significantly higher HBsAg and anti-HCV positivity in 2023 and 2024 compared to 2018, this may partly reflect changes in service delivery and testing patterns following the COVID-19 pandemic. Large surveys of liver centers have reported substantial reductions across the hepatitis care cascade during 2020, including marked decreases in HBV marker testing and HCV RNA testing, referrals, and treatment initiation, consistent with delayed diagnosis and a backlog of undetected infections [17]. In addition, interrupted time-series data from British Columbia showed that HBV screening and monitoring tests dropped early in the pandemic and remained below predicted levels through 2021–2022, supporting prolonged disruption rather than stable year-to-year ascertainment [18].

Geographic heterogeneity in viral hepatitis has been documented in Greece. For hepatitis B, studies from Central Greece (Thessaly) have identified municipal-level clustering and substantial variation between neighboring areas, highlighting localized transmission dynamics [8]. Similarly, national reviews of hepatitis C epidemiology describe regional variability across mainland and island populations, with evidence of intra-regional clustering rather than uniform distribution. These differences have been attributed to historical transmission patterns, migration flows, urban–rural population structure, and variation in healthcare access and testing practices [5]. In this context, the higher adjusted odds for HBsAg and anti-HCV positivity observed in Attica in our study could reflect the metropolitan concentration of migrant and vulnerable populations, as well as greater diagnostic activity in the capital region, rather than a uniform national pattern.

In our study, significantly higher odds of HBsAg positivity were observed among individuals originating from Albania and Pakistan, while lower odds were observed, among others, among those originating from Georgia, Bangladesh, and Iraq, when compared with Greece. For hepatitis C, higher odds of anti-HCV positivity were observed, among others, among individuals originating from Albania and Ethiopia. Published country-level prevalence estimates indicate that several of the aforementioned countries of origin report higher prevalence of HBsAg and/or anti-HCV compared with Greece [19]. Therefore, the elevated odds observed in our analysis for individuals originating from Albania and Pakistan (HBsAg), and from Albania and Ethiopia (anti-HCV), are consistent with existing epidemiological data [19]. In contrast, the significantly lower odds of HBsAg positivity among individuals originating from Georgia, Bangladesh, and Iraq, despite higher reported country-level prevalence compared with Greece, likely reflects differences between national prevalence estimates and the populations captured in this study. Previous evidence strongly suggests that hepatitis B prevalence among refugee and migrant populations may be lower than that reported for resident populations in countries of origin, potentially due to selection effects, pre-migration or post-arrival health screening, and differential access to healthcare [19]. Furthermore, the study population does not represent a general population sample, which further limits the direct comparability of our findings with national prevalence estimates. Together, these factors may account for the heterogeneity observed across countries of origin.

We found that several key populations had a higher prevalence of anti-HCV than the general population, consistent with prior evidence showing that groups such as people who inject drugs, people in prison, MSM (particularly in networks with overlapping risks), and some migrant subgroups experience a disproportionate burden of HCV exposure and ongoing transmission risk [20,21]. This aligns with systematic reviews from the EU/EEA reporting elevated HCV prevalence and, in some settings, measurable incidence in these populations, supporting their prioritization for targeted testing and prevention [22]. In contrast, we observed comparatively lower HBsAg prevalence among these populations, which have previously been identified as high-risk for HBsAg [22]. This difference may be partly attributable to methodological aspects of the HBsAg analysis. In particular, the HBsAg model was run after exclusion of vaccinated individuals, which may have modified the composition of the analytic population and the risk profile of the reference category. As a result, the reference group may have included individuals with unrecognized or unmeasured high-risk exposures who did not fall within the predefined key population categories but nevertheless contributed to HBsAg prevalence in the reference group. Moreover, this finding may reflect the nature of the study sample, which was drawn from individuals attending the hospital for various health-related reasons and therefore may not fully capture broader population-level prevalence.

This study has several limitations. First, the analysis is based on data from a single tertiary care hospital in the metropolitan area of Athens, and the hospital-based sampling frame—including both routine attendees and high-risk groups—limits the generalizability of the findings to the broader Greek population, particularly to rural regions or settings with different demographic and healthcare-access profiles. This design may also lead to an overestimation of seroprevalence compared with population-based estimates, as individuals with underlying medical conditions and socially vulnerable or high-risk groups are likely overrepresented. In addition, hepatitis B vaccination status was obtained through self-report in the absence of a national vaccination registry, introducing the possibility of misclassification. Furthermore, the restriction of the HBsAg regression analysis to self-reported unvaccinated individuals may have introduced bias. Vaccinated individuals may still be susceptible to infection or may have been incorrectly classified, which could influence the observed associations and limit comparability with the anti-HCV analysis. Moreover, the repeated cross-sectional design does not allow for causal inference or assessment of individual-level infection dynamics over time, and observed temporal trends may partly reflect changes in testing practices, healthcare access, or service disruptions rather than true changes in incidence or prevalence.

Overall, our findings highlight substantial heterogeneity and a persistently high clinical burden of HBV and HCV in this hospital-based population, with clear disparities by sex, geography, time period, and vulnerability status. These results reinforce the importance of timely diagnosis, effective treatment, and sustained follow-up to reduce morbidity and mortality and to prevent long-term complications.

## Figures and Tables

**Table 1:** Univariate and multivariable logistic regression analysis of factors associated with anti-HCV seropositivity.

Variable Name	Categories	OR (95% CI)	*p* Value	aOR (95% CI)	*p* Value
Gender	Female vs. Male	**0.78 (0.71–0.85)**	**<0.001**	**0.77 (0.70–0.84)**	**<0.001**
Age Group (ref. 18–29)	30–39	**0.94 (0.83–1.06)**	**0.306**	**0.79 (0.69–0.91)**	**0.001**
Age Group	40–49	**0.57 (0.50–0.65)**	**<0.001**	**0.58 (0.50–0.67)**	**<0.001**
Age Group	50–59	**0.52 (0.45–0.60)**	**<0.001**	**0.52 (0.45–0.60)**	**<0.001**
Age Group	60–69	**0.43 (0.37–0.49)**	**<0.001**	**0.41 (0.35–0.48)**	**<0.001**
Age Group	≥70	**0.27 (0.23–0.32)**	**<0.001**	**0.24 (0.20–0.29)**	**<0.001**
Nationality (ref. Greek)	Foreign	1.01 (0.92–1.10)	0.884	—	—
Country (ref. Greece)	Albania	**1.45 (1.20–1.75)**	**<0.001**	**1.43 (1.17–1.74)**	**<0.001**
Country	Afghanistan	1.22 (1.00–1.50)	0.052	1.12 (0.91–1.38)	0.292
Country	Pakistan	0.96 (0.73–1.25)	0.755	0.80 (0.61–1.06)	0.116
Country	Syria	**0.60 (0.43–0.83)**	**0.002**	**0.52 (0.37–0.72)**	**<0.001**
Country	Somalia	**0.66 (0.47–0.94)**	**0.020**	**0.59 (0.41–0.84)**	**0.003**
Country	Iraq	0.75 (0.54–1.04)	0.081	**0.69 (0.49–0.97)**	**0.032**
Country	Iran	0.76 (0.54–1.06)	0.107	**0.71 (0.50–1.00)**	**0.050**
Country	Morocco	0.83 (0.57–1.19)	0.313	0.86 (0.59–1.25)	0.423
Country	Ethiopia	**1.94 (1.50–2.52)**	**<0.001**	**2.15 (1.63–2.83)**	**<0.001**
Country	Egypt	1.11 (0.80–1.53)	0.544	1.19 (0.85–1.67)	0.314
Country	Bangladesh	0.91 (0.64–1.30)	0.600	0.93 (0.65–1.35)	0.713
Country	Philippines	1.00 (0.71–1.40)	0.989	1.05 (0.74–1.50)	0.785
Country	Georgia	1.09 (0.78–1.52)	0.604	1.22 (0.87–1.72)	0.248
Country	Ukraine	1.02 (0.69–1.53)	0.909	1.22 (0.81–1.86)	0.342
Country	Russia	1.19 (0.82–1.74)	0.362	**1.51 (1.02–2.23)**	**0.041**
Country	Senegal	0.81 (0.52–1.27)	0.361	1.01 (0.64–1.60)	0.959
Country	India	0.73 (0.46–1.16)	0.188	0.86 (0.53–1.41)	0.560
Country	Moldova	0.70 (0.43–1.13)	0.141	0.82 (0.50–1.36)	0.446
Country	China	1.08 (0.73–1.60)	0.699	1.37 (0.91–2.07)	0.129
Country	South Africa	0.96 (0.64–1.46)	0.862	1.20 (0.78–1.85)	0.400
Country	Turkey	1.05 (0.70–1.56)	0.823	1.24 (0.82–1.88)	0.310
Country	Bulgaria	1.27 (0.88–1.84)	0.207	**1.51 (1.02–2.22)**	**0.037**
Country	Romania	0.88 (0.57–1.36)	0.567	1.07 (0.68–1.67)	0.768
Country	Palestine	0.80 (0.51–1.25)	0.325	0.96 (0.60–1.53)	0.852
Area (ref. Attica)	Peloponnese	**0.82 (0.71–0.94)**	**0.005**	**0.81 (0.70–0.93)**	**0.004**
Area	Thessaly	**1.20 (1.05–1.38)**	**0.009**	**1.19 (1.03–1.37)**	**0.017**
Area	Macedonia	**0.66 (0.54–0.80)**	**<0.001**	**0.64 (0.52–0.78)**	**<0.001**
Area	Thrace	**0.36 (0.23–0.55)**	**<0.001**	**0.32 (0.21–0.50)**	**<0.001**
Area	Cycladic Islands	**0.37 (0.30–0.47)**	**<0.001**	**0.35 (0.28–0.45)**	**<0.001**
Area	Dodecanese Islands	**0.34 (0.27–0.44)**	**<0.001**	**0.32 (0.25–0.41)**	**<0.001**
Area	Sporades	**0.36 (0.23–0.55)**	**<0.001**	**0.33 (0.21–0.51)**	**<0.001**
Area	Ionian Islands	**0.64 (0.45–0.89)**	**0.008**	**0.57 (0.40–0.80)**	**0.001**
Area	Crete	**0.38 (0.27–0.54)**	**<0.001**	**0.35 (0.24–0.50)**	**<0.001**
Area	Euboea	**0.35 (0.24–0.51)**	**<0.001**	**0.34 (0.23–0.50)**	**<0.001**
Year (ref. 2018)	2019	0.95 (0.80–1.13)	0.552	0.95 (0.80–1.13)	0.590
Year	2020	1.17 (0.99–1.38)	0.058	**1.20 (1.01–1.42)**	**0.037**
Year	2021	1.00 (0.84–1.19)	0.996	1.00 (0.84–1.19)	0.994
Year	2022	1.17 (1.00–1.38)	0.054	1.16 (0.99–1.38)	0.075
Year	2023	**1.35 (1.15–1.59)**	**<0.001**	**1.38 (1.17–1.63)**	**<0.001**
Year	2024	**1.71 (1.46–1.99)**	**<0.001**	**1.78 (1.52–2.09)**	**<0.001**
HepB vaccination (ref. No)	Yes	**0.40 (0.36–0.44)**	**<0.001**	1.27 (0.97–1.67)	0.087
Underlying condition	Cardiovascular disease	**0.61 (0.42–0.89)**	**0.010**	**2.01 (1.34–3.03)**	**0.001**
Underlying condition	Diabetes mellitus	**0.37 (0.28–0.50)**	**<0.001**	**0.63 (0.47–0.86)**	**0.003**
Underlying condition	Autoimmune diseases	**1.93 (1.63–2.27)**	**<0.001**	**1.75 (1.43–2.13)**	**<0.001**
Underlying condition	Neurological diseases	0.95 (0.74–1.22)	0.683	**2.02 (1.53–2.68)**	**<0.001**
Underlying condition	Lipid disorders	**0.51 (0.43–0.62)**	**<0.001**	**0.68 (0.55–0.83)**	**<0.001**
Social group	Substance users (ex and current)	**3.52 (2.96–4.18)**	**<0.001**	**4.68 (3.45–6.32)**	**<0.001**
Social group	MSM	**4.53 (3.54–5.80)**	**<0.001**	**5.51 (3.85–7.89)**	**<0.001**
Social group	Roma	**3.83 (3.39–4.33)**	**<0.001**	**4.98 (3.79–6.55)**	**<0.001**
Social group	Refugees	**4.05 (3.43–4.77)**	**<0.001**	**5.27 (3.91–7.09)**	**<0.001**
Social group	Immigrants	**3.01 (2.51–3.61)**	**<0.001**	**3.90 (2.87–5.31)**	**<0.001**
Social group	Incarcerated people	**4.08 (3.61–4.60)**	**<0.001**	**5.41 (4.11–7.12)**	**<0.001**
Social group	Sex workers	**3.11 (2.49–3.88)**	**<0.001**	**4.59 (3.28–6.41)**	**<0.001**

OR: odds ratio; aOR: adjusted odds ratio; 95% CI: 95% confidence interval. Values in bold indicate statistical significance (*p* < 0.05).

**Table 2:** Univariate and multivariable logistic regression analysis of factors associated with HBsAg seropositivity.

Variable Name	Categories	OR (95% CI)	*p* Value	aOR (95% CI)	*p* Value
Sex	Female vs. Male	**0.28 (0.25–0.32)**	**<0.001**	**0.24 (0.21–0.27)**	**<0.001**
Age Group (ref. 18–29)	30–39	0.96 (0.79–1.16)	0.681	1.10 (0.89–1.35)	0.380
Age Group	40–49	0.88 (0.72–1.07)	0.200	1.05 (0.85–1.29)	0.679
Age Group	50–59	0.83 (0.68–1.01)	0.062	**0.75 (0.61–0.93)**	**0.008**
Age Group	60–69	**0.54 (0.43–0.66)**	**<0.001**	**0.49 (0.40–0.62)**	**<0.001**
Age Group	≥70	1.15 (0.98–1.34)	0.085	**0.75 (0.61–0.92)**	**0.005**
Nationality (ref. Greek)	Foreign	**0.90 (0.81–0.99)**	**0.039**	—	—
Country (ref. Greece)	Albania	**1.93 (1.59–2.36)**	**<0.001**	**1.91 (1.54–2.37)**	**<0.001**
Country	Afghanistan	1.09 (0.86–1.39)	0.481	1.05 (0.81–1.35)	0.732
Country	Pakistan	**1.55 (1.20–2.02)**	**0.001**	**1.55 (1.17–2.05)**	**0.002**
Country	Syria	0.77 (0.55–1.08)	0.128	0.71 (0.50–1.02)	0.061
Country	Somalia	0.72 (0.49–1.05)	0.091	0.69 (0.47–1.04)	0.079
Country	Iraq	**0.58 (0.38–0.88)**	**0.011**	**0.58 (0.38–0.90)**	**0.015**
Country	Iran	**0.65 (0.43–0.98)**	**0.039**	0.66 (0.43–1.00)	0.056
Country	Morocco	0.68 (0.43–1.08)	0.104	0.71 (0.44–1.15)	0.167
Country	Ethiopia	0.89 (0.59–1.34)	0.566	0.95 (0.61–1.47)	0.817
Country	Egypt	1.03 (0.70–1.52)	0.876	1.14 (0.76–1.71)	0.520
Country	Bangladesh	**0.56 (0.34–0.94)**	**0.027**	**0.59 (0.35–0.99)**	**0.046**
Country	Philippines	**0.57 (0.34–0.94)**	**0.030**	**0.57 (0.34–0.96)**	**0.034**
Country	Georgia	**0.52 (0.31–0.88)**	**0.016**	**0.49 (0.29–0.85)**	**0.010**
Country	Ukraine	0.77 (0.45–1.31)	0.342	0.76 (0.43–1.35)	0.349
Country	Russia	0.59 (0.33–1.06)	0.078	0.58 (0.31–1.10)	0.096
Country	Senegal	0.64 (0.37–1.13)	0.126	0.65 (0.36–1.20)	0.169
Country	India	0.58 (0.32–1.05)	0.070	0.55 (0.29–1.04)	0.067
Country	Moldova	**0.28 (0.12–0.63)**	**0.002**	**0.24 (0.10–0.59)**	**0.002**
Country	China	**0.44 (0.22–0.86)**	**0.016**	**0.47 (0.23–0.97)**	**0.041**
Country	South Africa	**0.54 (0.29–0.99)**	**0.049**	0.60 (0.31–1.16)	0.127
Country	Turkey	0.77 (0.46–1.31)	0.342	0.92 (0.53–1.60)	0.760
Country	Bulgaria	0.77 (0.45–1.30)	0.322	0.94 (0.54–1.63)	0.813
Country	Romania	0.86 (0.52–1.42)	0.563	1.01 (0.59–1.72)	0.969
Country	Palestine	1.28 (0.84–1.94)	0.259	1.50 (0.95–2.36)	0.083
Area (ref. Attica)	Peloponnese	0.88 (0.76–1.03)	0.106	0.95 (0.81–1.11)	0.480
Area	Thessaly	**0.23 (0.17–0.31)**	**<0.001**	**0.21 (0.15–0.28)**	**<0.001**
Area	Macedonia	**0.36 (0.27–0.47)**	**<0.001**	**0.37 (0.28–0.50)**	**<0.001**
Area	Thrace	**0.08 (0.03–0.21)**	**<0.001**	**0.08 (0.03–0.20)**	**<0.001**
Area	Cycladic Islands	**0.35 (0.27–0.45)**	**<0.001**	**0.30 (0.23–0.40)**	**<0.001**
Area	Dodecanese Islands	**0.34 (0.26–0.45)**	**<0.001**	**0.29 (0.22–0.38)**	**<0.001**
Area	Sporades	**0.10 (0.04–0.24)**	**<0.001**	**0.09 (0.04–0.22)**	**<0.001**
Area	Ionian Islands	**0.25 (0.14–0.44)**	**<0.001**	**0.19 (0.11–0.34)**	**<0.001**
Area	Crete	**0.14 (0.08–0.27)**	**<0.001**	**0.13 (0.07–0.24)**	**<0.001**
Area	Euboea	**0.18 (0.10–0.32)**	**<0.001**	**0.18 (0.10–0.33)**	**<0.001**
Year (ref. 2018)	2019	0.95 (0.77–1.18)	0.646	0.95 (0.75–1.18)	0.609
Year	2020	**1.24 (1.00–1.53)**	**0.048**	1.21 (0.97–1.50)	0.095
Year	2021	1.18 (0.96–1.46)	0.119	1.20 (0.96–1.49)	0.109
Year	2022	**1.28 (1.04–1.58)**	**0.018**	**1.27 (1.02–1.57)**	**0.029**
Year	2023	**1.49 (1.22–1.83)**	**<0.001**	**1.51 (1.22–1.86)**	**<0.001**
Year	2024	**2.76 (2.29–3.32)**	**<0.001**	**3.10 (2.55–3.77)**	**<0.001**
Underlying Condition (ref. None)	Cardiovascular Disease	0.88 (0.64–1.20)	0.427	**0.59 (0.42–0.83)**	**0.002**
Underlying Condition	Diabetes mellitus	**1.92 (1.59–2.31)**	**<0.001**	**2.72 (2.20–3.37)**	**<0.001**
Underlying Condition	Autoimmune Diseases	**0.62 (0.41–0.92)**	**0.018**	**0.47 (0.31–0.73)**	**0.001**
Underlying Condition	Neurological diseases	**2.28 (1.86–2.78)**	**<0.001**	**2.09 (1.65–2.64)**	**<0.001**
Underlying Condition	Lipid disorders	**0.53 (0.42–0.67)**	**<0.001**	**0.45 (0.35–0.58)**	**<0.001**
Social Group (ref. Not in a social minority)	Substance users (Ex and current)	**0.68 (0.55–0.83)**	**<0.001**	**0.55 (0.43–0.70)**	**<0.001**
Social Group	MSM	**0.63 (0.45–0.86)**	**0.004**	**0.32 (0.22–0.46)**	**<0.001**
Social Group	Roma	**0.66 (0.57–0.77)**	**<0.001**	**0.53 (0.44–0.65)**	**<0.001**
Social Group	Refugees	**0.73 (0.60–0.89)**	**0.002**	**0.59 (0.46–0.75)**	**<0.001**
Social Group	Immigrants	**0.71 (0.58–0.86)**	**0.001**	**0.57 (0.45–0.72)**	**<0.001**
Social Group	Incarcerated people	**0.75 (0.65–0.86)**	**<0.001**	**0.60 (0.49–0.73)**	**<0.001**
Social Group	Sex workers	**0.52 (0.40–0.68)**	**<0.001**	**0.60 (0.44–0.82)**	**0.001**

Model restricted to unvaccinated participants (n = 18,719). OR: odds ratio; aOR: adjusted odds ratio; 95% CI: 95% confidence interval. Values in bold indicate statistical significance (*p* < 0.05).

## Data Availability

Data would become available upon reasonable request.

## Supplementary Materials

The following supporting information can be downloaded at: https://www.mdpi.com/article/10.3390/pathogens15030342/s1, Figure S1: Annual trend in anti-HCV seropositivity in the study population, 2018–2024; Figure S2: Annual trend in anti-HCV seropositivity stratified by sex, 2018–2024; Figure S3: Annual trend in HBsAg seropositivity among unvaccinated individuals, 2018–2024; Figure S4: Annual trend in HBsAg seropositivity among unvaccinated individuals stratified by sex, 2018–2024.
