# Supplementary material for: HBV and HCV Burden in a Greek Hospital Population (2018–2024): Trends and Correlates of HBsAg and Anti-HCV Positivity
Source: Pathogens. 2026 Mar 23;15(3):342. doi: 10.3390/pathogens15030342 (PMC13029126; doi:10.3390/pathogens15030342)
Supplement: Supplementary file 1 [file pathogens-15-00342-s001.zip › pathogens-4222608-supplementary.pdf]

Supplemental Material

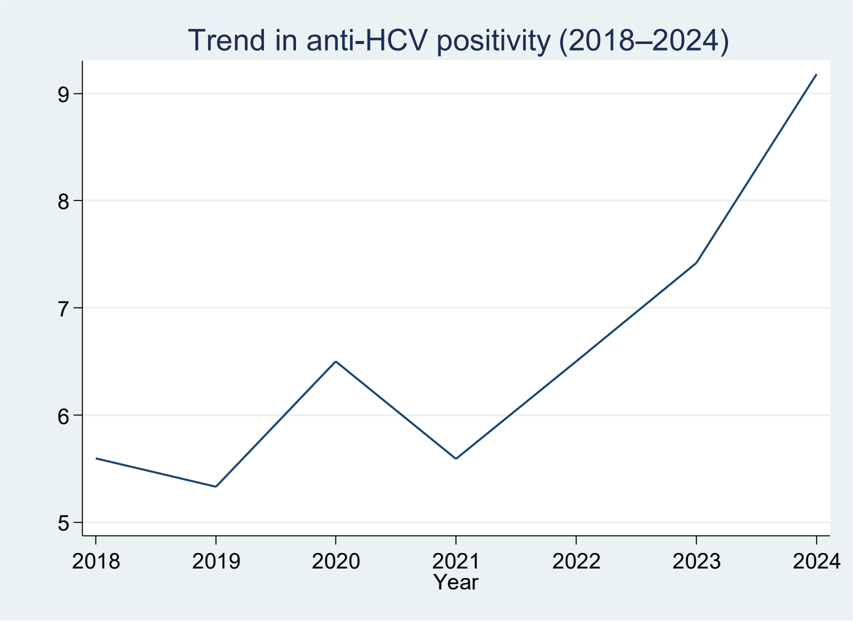

Anti-HCV seropositivity is expressed as the percentage of individuals testing positive among those tested each year.

Supplemental Figure S1. "Annual trend in anti-HCV seropositivity in the study population, 2018–2024".

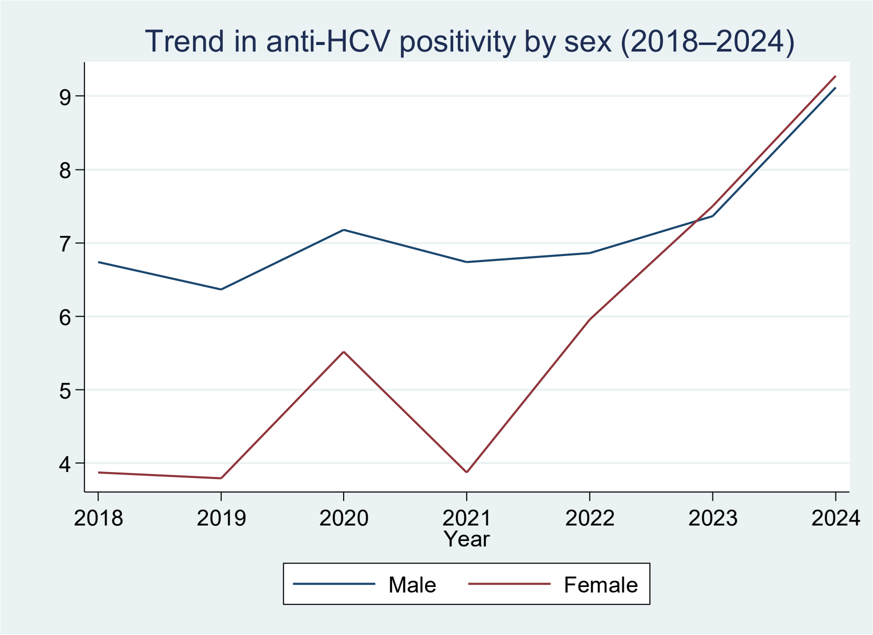

Anti-HCV seropositivity is expressed as the percentage of individuals testing positive among those tested each year. Estimates are presented separately for males and females.

Supplemental Figure S2. "Annual trend in anti-HCV seropositivity stratified by sex, 2018–2024".

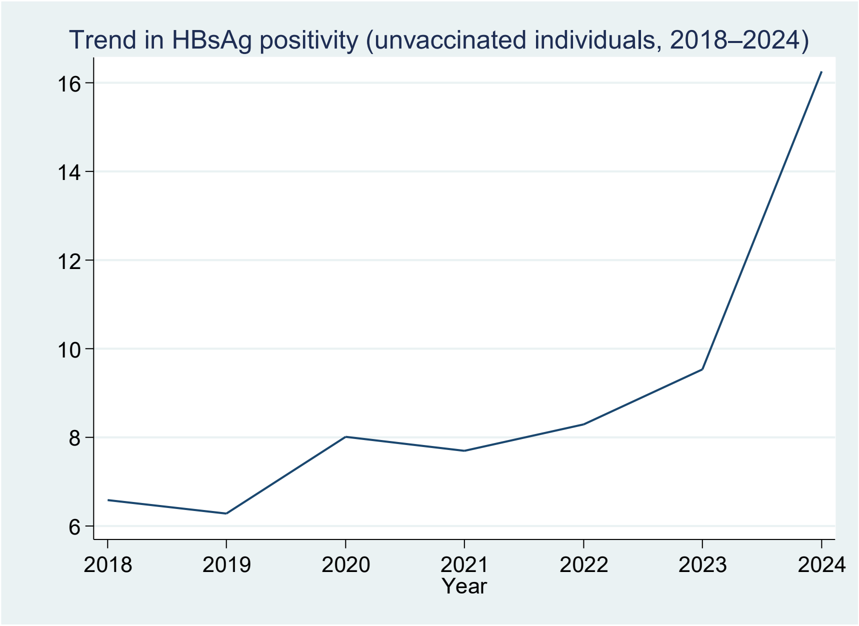

HBsAg seropositivity is expressed as the percentage of individuals testing positive among those tested each year. Analysis restricted to individuals who self-reported not being vaccinated against hepatitis B.

Supplemental Figure S3. "Annual trend in HBsAg seropositivity among unvaccinated individuals, 2018–2024".

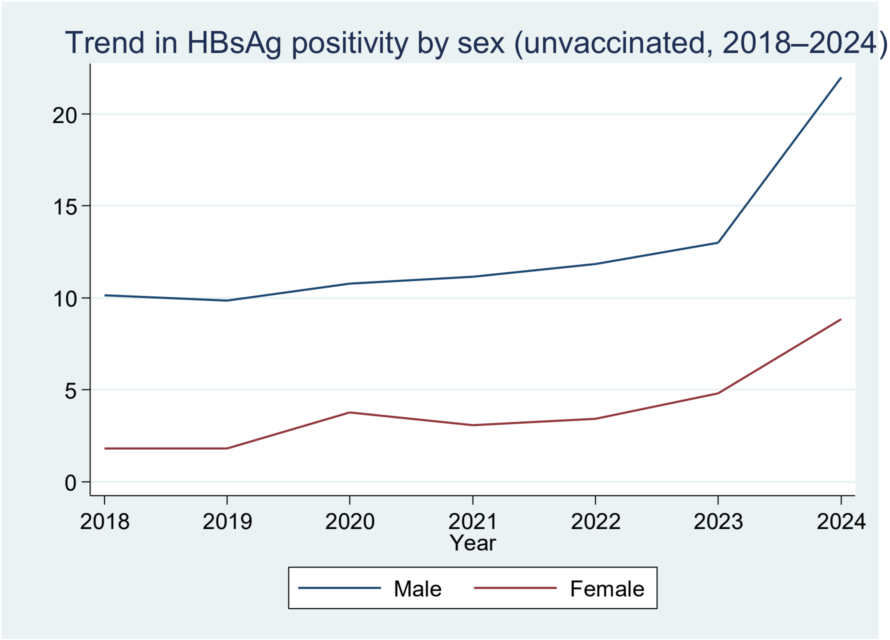

HBsAg seropositivity is expressed as the percentage of individuals testing positive among those tested each year. Analysis restricted to individuals who self-reported not being vaccinated against hepatitis B. Estimates are presented separately for males and females

Supplemental Figure S4. "Annual trend in HBsAg seropositivity among unvaccinated individuals stratified by sex, 2018–2024".
